# Silver Nanoparticles for Biosensing and Drug Delivery: A Mechanical Study on DNA Interaction

**DOI:** 10.3390/bios15050331

**Published:** 2025-05-21

**Authors:** Katarína Nemčeková, Patrícia Dudoňová, Tomáš Holka, Sabína Balážová, Michaela Hornychová, Viktória Szebellaiová, Monika Naumowicz, Pavol Gemeiner, Tomáš Mackuľak, Miroslav Gál, Veronika Svitková

**Affiliations:** 1 Department of Inorganic Technology, Faculty of Chemical and Food Technology, Slovak University of Technology in Bratislava, 812 37 Bratislava, Slovakia; katarina.nemcekova@stuba.sk (K.N.); michaela.hornychova@stuba.sk (M.H.); viktoria.szebellaiova@stuba.sk (V.S.); 2 Institute of Analytical Chemistry, Faculty of Chemical and Food Technology, Slovak University of Technology in Bratislava, 812 37 Bratislava, Slovakia; chempdud@savba.sk (P.D.); xholka@stuba.sk (T.H.); xbalazovas@stuba.sk (S.B.); 3 Institute of Chemistry, Slovak Academy of Sciences, 845 38 Bratislava, Slovakia; 4 Department of Physical Chemistry, Faculty of Chemistry, University of Bialystok, 15-245 Bialystok, Poland; monikan@uwb.edu.pl; 5 Department of Graphic Arts Technology and Applied Photochemistry, Faculty of Chemical and Food Technology, Slovak University of Technology in Bratislava, Radlinského 9, 812 37 Bratislava, Slovakia; pavol.gemeiner@stuba.sk; 6 Department of Environmental Engineering, Faculty of Chemical and Food Technology, Slovak University of Technology in Bratislava, 812 37 Bratislava, Slovakia; tomas.mackulak@stuba.sk; 7 MicroPoll s.r.o., Vazovova 5, 812 43 Bratislava, Slovakia

**Keywords:** silver nanoparticles, targeted drug delivery, biosensors, nanocarriers, doxorubicin, oxidative stress, green synthesis, cancer

## Abstract

Silver nanoparticles (AgNPs) have attracted tremendous attention in recent years due to their unique physicochemical properties, including pronounced surface plasmon resonance, tunable size, and amenability to functionalization. These attributes underpin the growing interest in AgNPs as SMART nanocarriers for targeted drug delivery and as active components in biosensing platforms. In this work, we discuss various synthesis strategies for AgNPs—ranging from conventional chemical methods to green approaches—and highlight their subsequent functionalization with anticancer drugs, notably doxorubicin (DOX). We also examine the potential of AgNPs in biosensor applications, emphasizing electrochemical and optical detection modalities capable of monitoring drug release, oxidative stress, and relevant biomarkers. Our experimental data support the conclusion that AgNPs can effectively improve therapeutic efficacy by exploiting tumor-specific conditions (e.g., lower pH) while also enhancing biosensor sensitivity via surface plasmon resonance and electrochemical signal amplification. We provide a thorough discussion of the results, including mechanistic aspects of reactive oxygen species (ROS) generation, drug release kinetics, and sensor performance metrics. Overall, AgNP-based nanocarriers emerge as a powerful platform to address current challenges in precision oncology and medical diagnostics.

## 1. Introduction

Nanotechnology is recognized as a rapidly growing field that integrates principles from chemistry, physics, biology, and engineering to develop materials and devices at the nanoscale. Among the various categories of nanostructures, silver nanoparticles (AgNPs) have attracted significant interest, e.g., for biomedical applications. Increased interest in AgNPs is due to several factors: they demonstrate distinctive optical and electrical characteristics, with a significant surface plasmon resonance (SPR) band that can be modulated by varying particle size, shape, and local environment [1,2]. Second, they possess favorable chemical reactivity and can be relatively easily surface-modified to carry drugs, targeting ligands, or other functional moieties [3]. Third, AgNPs have well-documented antibacterial, antifungal, and antiviral potential, which favor them for long-term use in wound dressings and related applications [4,5]. More recently, silver nanoparticles have been explored for antitumor capabilities and synergy with chemotherapeutic agents, enabling them to serve as “SMART” nanocarriers in targeted oncology therapies [6,7]. However, upon ingestion, AgNPs are easily absorbed by the gastrointestinal tract, enter the circulatory system, and are accumulated in the brain, lungs, liver, kidneys, and testes. Prolonged exposure can also lead to organ-specific toxicity and inflammatory reactions [8].

The concept of using metal nanoparticles as drug delivery vehicles or as imaging contrast agents is not a novel one; nevertheless, silver continues to be a subject of considerable interest. The reasons for this are threefold: firstly, its cost-effectiveness when compared with gold; secondly, its robust plasmonic properties; and thirdly, its potential to be synthesized under relatively mild and environmentally friendly conditions. Several studies have reported on “green” routes for the synthesis of AgNPs that rely on natural reductants (e.g., gallic acid, plant extracts, or polysaccharides), thus minimizing the use of toxic reagents like sodium borohydride (NaBH4) and making the entire process more sustainable [9,10]. Green-synthesized AgNPs frequently demonstrate favorable biocompatibility and minimal unwanted side effects, which is important for biomedical applications where toxicity remains a major concern [11].

Cancer therapy is an especially promising area for AgNP applications. Among lifestyle-related diseases, cancer is one of the most serious, being the second most common cause of death worldwide. In 2020, it was diagnosed in nearly 3 million people in the European Union and claimed 1.27 million lives (europarl.europa.eu), and the number of deaths is projected to rise by 47% by 2040 [12]. Conventional anticancer treatments, such as systemic chemotherapy, often result in off-target toxicity due to the non-specific distribution of potent drugs like doxorubicin (DOX). These adverse effects compromise patient outcomes and limit therapeutic dosages [13]. Therefore, the design of nanoparticles capable of responding to or exploiting specific tumor microenvironment triggers—like low pH, hypoxia, or high oxidative stress—remains at the forefront of nanomedicine research [6,14]. AgNPs can be engineered to integrate seamlessly with such triggers. For instance, acidic pH (close to 5–6) in tumor niches can enhance the release of a conjugated chemotherapeutic from AgNP surfaces, minimizing systemic exposure and maximizing impact on malignant cells [15]. Furthermore, partial dissolution of AgNPs can lead to the generation of Ag^+^ ions, which may act synergistically with the drug to induce cytotoxicity or modulate oxidative stress pathways [16,17]. Transferrin-conjugated magnetic dextran-spermine nanoparticles have shown efficacy in targeted drug transport across the blood–brain barrier, thereby opening new opportunities for the treatment of central nervous system disorders [18]. Similarly, folate receptor-targeted multimodal polymersomes have been effectively employed to co-deliver quantum dots and the anticancer drug doxorubicin, resulting in enhanced therapeutic efficacy against breast adenocarcinoma in both in vitro and in vivo models [19]. Silk fibroin nanoparticles are another promising platform, as they are known for their biodegradability, biocompatibility, and tunable release properties, which are advantageous in controlled drug delivery applications [20]. Aptamer-conjugated quantum dots, particularly those modified with AS1411 aptamers, exhibit high fluorescence and specificity in labeling and imaging tumor cells, further increasing their utility in diagnostic imaging [21]. Furthermore, polymeric nanoparticles remain a subject of extensive exploration due to their versatility in simultaneous therapy and imaging, enabling the development of theranostic platforms for personalized medicine [22]. Polyrotaxane-capped quantum dots have recently attracted significant attention due to their potential as novel diagnostic and therapeutic tools, offering superior stability and targeting capabilities [23].

Another important area is the use of AgNPs in biosensor technology. The capacity of AgNPs to enhance electron-transfer processes and amplify optical signals has opened pathways for high-sensitivity detection of biomarkers associated with oxidative stress, cancer progression, and pathogen presence [24,25]. The synergy between AgNP-based drug delivery and biosensor platforms could catalyze the emergence of integrated theranostic systems. In such systems, nanoparticles do not merely deliver a therapeutic agent but also facilitate real-time monitoring of biological responses, for example, by detecting reactive oxygen species (ROS) or shifts in pH [26]. Electrochemical biosensors, in particular, stand out for their adaptability, low cost, and ease of miniaturization, enabling the creation of portable “point-of-care” (POC) devices [27,28]. When AgNPs are immobilized on an electrode surface, they can boost electron-transfer kinetics, thereby improving sensitivity to analytes like H_2_O_2_ or superoxide radicals. Meanwhile, surface-enhanced Raman scattering (SERS) can be used to analyze molecular species in extremely low concentrations, capitalizing on the strong electromagnetic fields that form near AgNPs [29,30].

Despite the immense promise, there remain a few critical challenges. One major obstacle is the reproducible large-scale synthesis of AgNPs with well-defined size and shape distributions, which is essential for consistent drug-loading and release profiles, as well as for uniform sensor responses [31]. Another challenge pertains to potential cytotoxicity and environmental accumulation; while AgNPs are generally considered more biocompatible than certain other metal nanoparticles, their safety profile at higher doses or upon prolonged exposure is still debated [32]. Efforts in “green” synthesis and surface functionalization with biopolymers aim to mitigate these concerns, but further in vitro and in vivo studies are needed. Additionally, the synergy between drug release and the intrinsic antibacterial or pro-oxidative effects of AgNPs may be beneficial in certain contexts (e.g., infective complications in cancer therapy) but requires careful calibration to avoid damage to healthy tissues or off-target organs.

This paper builds upon previous research [33], which has elucidated mechanistic aspects of how AgNPs can be formulated into “SMART” nanosystems for drug delivery and used as perspective carriers for doxorubicin. We integrate those findings into the broader context of biosensor design, particularly emphasizing how changes in physiochemical conditions (pH, presence of ROS) can be monitored electrochemically or via the spectroscopic methods. We begin by detailing the experimental protocols used to synthesize and characterize AgNPs (Section 2), focusing on chemical, green, and seed-mediated growth approaches. Subsequently, in Section 3, we present results that highlight the dual potential of these AgNPs for controlled DOX release in tumor-like environments and for biosensing applications, with special attention to electrochemical measurements. Finally, we discuss the broader implications of this technology and outline avenues for future work (Section 4), concluding with an overview of the potential role that AgNP-based hybrid systems may play in addressing critical challenges in oncology and diagnostics (Section 5).

## 2. Materials and Methods

### 2.1. Chemicals and Reagents

Silver nitrate (AgNO_3_, ≥99.9%) was purchased from Sigma-Aldrich (Taufkirchen, Germany). Sodium borohydride (NaBH_4_, 98%), polyvinylpyrrolidone (PVP, average Mw ~40,000), and hexadecyltrimethylammonium bromide (CTAB, ≥99%) were obtained from Merck (Bratislava, Slovakia). Gallic acid (≥97%) for the green synthesis was sourced from Sigma-Aldrich (Bratislava, Slovakia). Doxorubicin hydrochloride (DOX·HCl) was purchased from Sigma-Aldrich (Bratislava, Slovakia). Phosphate buffer solutions (PBSs) at pH 7.4 and 4.5, potassium ferricyanide ([Fe(CN)_6_]^3−^), and potassium ferrocyanide ([Fe(CN)_6_]^4−^) were from Centralchem (Bratislava, Slovakia). All solutions were prepared using deionized water (18.2 MΩ·cm resistivity). Prior to use in any experiments, glassware was washed with aqua regia (3:1 mixture of HCl and HNO_3_) and thoroughly rinsed with deionized water to avoid contamination. All experiments were carried out at room temperature (~25 °C) unless otherwise specified.

### 2.2. Synthesis of Silver Nanoparticles

#### 2.2.1. Chemical Reduction Approach

A conventional chemical reduction method was adopted using NaBH_4_, which reduces AgNO_3_ to elemental silver in the presence of stabilizers [34]. First, 10 mL of 1 mM AgNO_3_ was prepared. Separately, 30 mL of 2 mM NaBH_4_ solution was cooled in an ice bath for 15 min. The AgNO_3_ solution was then added dropwise (~1 drop/s) into the NaBH_4_ solution under vigorous stirring (600 rpm), maintaining the temperature between 0 and 5 °C. The reaction mixture changed from colorless to a light-yellow hue, indicating the formation of silver colloids. Next, 1–2 mL of PVP solution (10 g·L^−1^) was added to improve stability. The resulting silver nanoparticles (spherical, ~10–20 nm) were stored in dark vials at 4 °C until further use. UV-Vis spectra were recorded to confirm the SPR peak at ~420 nm.

#### 2.2.2. Green Synthesis with Gallic Acid

Inspired by the reference [35], we performed a green synthesis using gallic acid as both a reductant and a capping agent. Briefly, 0.01 g of gallic acid was dissolved in 10 mL of deionized water. Separately, 100 mL of 1 mM AgNO_3_ was adjusted to pH ~10 using ammonium hydroxide (NH_3_). The two solutions were combined and stirred at room temperature for 1 h. Over time, the reaction mixture turned yellowish-brown, indicating the formation of silver nanoparticles. To monitor progress, aliquots of the colloid were analyzed by UV-Vis, expecting a characteristic SPR band near 430 nm. Additionally, ATR-FTIR spectra were collected to identify shifts in gallic-acid-related peaks (such as O–H and C=O stretches), indicative of metal–ligand binding [36]. The final product contained predominantly spherical AgNPs (~15 nm average diameter, as validated by SEM and size distribution diagram). The SEM technique was utilized to characterize NPs with an accelerating voltage ranging from 20 to 25 kV for the samples that were prepared on pre-cleaned silicon oxide substrates and processed without prior metal sputtering. The size distribution diagram was subsequently plotted using an image editor, ImageJ 1.54. Single-drop ATR-FTIR analysis was performed on a diamond crystal ATR instrument, with spectra recorded between 4000 and 500 cm^−1^ using a Shimadzu IRSpirit-X FTIR spectrophotometer equipped with IR Pilot^TM^ software (Shimadzu Slovakia, Bratislava, Slovakia).

#### 2.2.3. Seed-Mediated Growth of Triangular Nanoprisms

To obtain anisotropic nanoparticles (nanoprism morphologies), a seed-mediated protocol was adapted from prior studies [37]. First, silver “seed” solutions (~5 nm seeds) were synthesized via a chemical reduction method at low temperature. In a separate step, 25 mL of 0.1 mM AgNO_3_, 1.5 mL of 30 mM sodium citrate, 1.5 mL of 0.7 mM polyvinylpyrrolidone (PVP), and 60 µL of 30% H_2_O_2_ were mixed and heated to 80 °C. After the mixture reached 80 °C, ~100 µL of the seed solution was injected, leading to an immediate color change from pale yellow to orange-red, then to a purple or blue hue indicating nanoprism formation. Samples were taken at intervals to measure SPR peaks in the 600–800 nm range. Triangular prisms typically exhibited a primary SPR at ~750 nm, along with weaker shoulder peaks. The reaction was halted by cooling the flask in an ice bath after ~20 min.

### 2.3. Functionalization with Doxorubicin (DOX)

**Figure 1:**
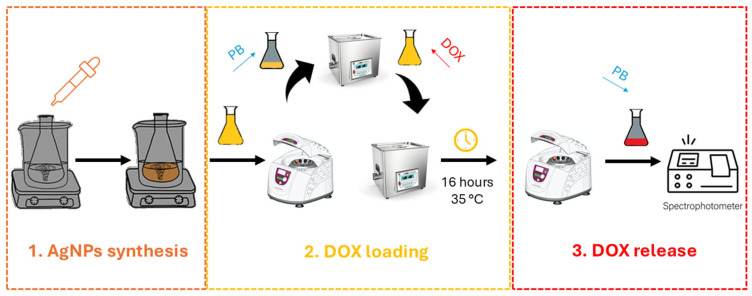
Schematic illustration of step-by-step workflow.

AgNPs were conjugated with doxorubicin via electrostatic interactions, hydrogen bonding, and, in some cases, hydrophobic effects depending on the surface capping layer [38]. To standardize conditions (Figure 1), an aliquot of the AgNP suspension (optical density ~1.0 at its peak) was centrifuged at 15,000 rpm for 15 min. The supernatant was removed, and the nanoparticle pellet was gently resuspended in 0.1 M PBS (pH 7.4 or 4.5). DOX (1 mg·mL^−1^ in water) was then added dropwise to the AgNP suspension to achieve a final doxorubicin concentration of 10–50 µg·mL^−1^. This mixture was kept under mild agitation (ultrasonication or gentle stirring) at 35 °C, away from light, for times ranging from 16 to 48 h. UV-Vis spectroscopy at 480–550 nm was used to track unbound DOX in the supernatant after centrifugation.

### 2.4. Biosensor Fabrication and Electrochemical Measurements

Glassy carbon electrodes (GCEs, 3 mm diameter) were first polished using 1.0 and 0.05 µm alumina slurry on Mohair paper, rinsed thoroughly with deionized water, and sonicated briefly to remove residual alumina. Electrochemical activation was performed by applying +1.5 V for 300 s in 0.1 M PBS (pH 7.4).

Double-stranded DNA (dsDNA) from salmon sperm (1 mg·mL^−1^) was immobilized using an electrostatic deposition approach. The GCE was placed in a cell containing a dsDNA solution in PBS, and a potential of +0.6 V was applied for 300 s. This step allowed dsDNA molecules to adhere to the positively charged electrode surface. The electrode was subsequently rinsed to remove loosely bound DNA.

Aliquots of AgNPs (spherical or nanoprism forms) were drop-cast onto the dsDNA/GCE, followed by 10–60 min of incubation. Afterward, the electrode was gently rinsed with water to remove unbound nanoparticles. Control electrodes (without AgNPs) were also prepared to distinguish the baseline responses of dsDNA layers alone.

Electrochemical impedance spectroscopy (EIS) was conducted in 1 mM [Fe(CN)_6_]^3−^/[Fe(CN)_6_]^4−^ containing 0.1 M KCl. A Metrohm Autolab potentiostat/galvanostat (PGSTAT12) recorded Nyquist plots at +0.25 V vs. Ag/AgCl from 100 kHz down to 0.1 Hz [3,14]. Cyclic voltammetry (CV) scanning from −0.4 V to +0.7 V at 100 mV·s^−1^ provided insights into electron-transfer changes caused by dsDNA and the AgNP layers. Differential pulse voltammetry (DPV) in 0.1 M PBS was used to detect adenine and guanine oxidation peaks at ~+1.0 and ~+0.75 V, respectively. Shifts in peak current or potential indicated interactions between AgNPs and dsDNA.

## 3. Results

### 3.1. Characterization of AgNPs

#### 3.1.1. UV-Vis Spectra and Particle Size

**Figure 2:**
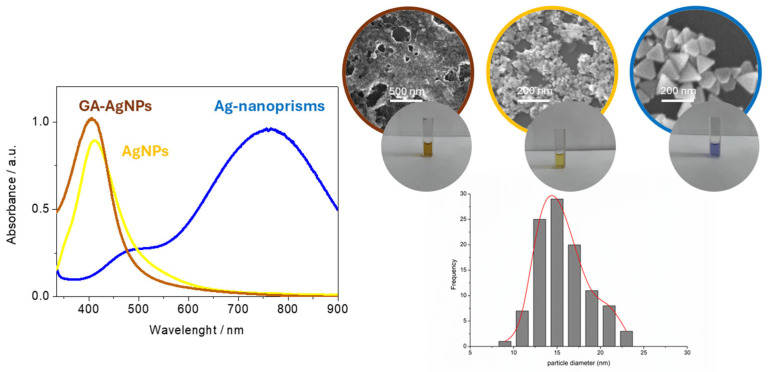
UV-Vis spectra and SEM images of synthesized AgNPs. Distribution diagram of GA-AgNPs.

All three synthesis routes yielded stable colloids with characteristic SPR peaks (Figure 2, summarized in Table 1). For chemically reduced NPs, the maximum absorbance (λmax) was around 420 nm (particles marked as AgNPs), in line with the formation of small, spherical-shaped particles. Green-synthesized AgNPs (marked as GA-AgNPs) showed a similar SPR at ~410 nm. In seed-mediated growth (marked as Ag-nanoprisms), larger and anisotropic, triangularly shaped prisms exhibited peaks above 600 nm, usually near 750 nm. UV-Vis spectral analysis was performed by spectrophotometer Thermo Scientific-Evolution 201 using 10 mm path quartz cuvettes.

Based on the SEM measurements, the average size for chemical-synthesized AgNPs was 10–20 nm, for green-synthesized AgNPs 15 ± 4 nm, and for triangular prisms 50–100 nm. ATR-FTIR analyses confirmed the presence of gallic acid moieties on the green-synthesized AgNP surface via strong absorption bands around 1710 cm^−1^ (C=O stretch) and ~1030 cm^−1^ (aromatic C–O) [36].

#### 3.1.2. Stability and pH Dependence

When stored in dark conditions at room temperature, both the chemically reduced and green-synthesized AgNPs maintained minimal aggregation for up to 14 days, as evidenced by minimal changes in their UV-Vis spectra. By day 14, the main SPR peak for the smaller, spherical AgNPs slightly decreased in intensity, indicating the onset of mild aggregation or Ostwald ripening [39,40]. Meanwhile, nanoprism solutions experienced a more pronounced change in color (blue to purple) after one to two weeks, reflecting their higher susceptibility to shape transformation or edge etching. Notably, solutions at pH < 5 often accelerated morphological changes, which can be leveraged in pH-sensitive cargo release contexts [41].

### 3.2. Doxorubicin Loading and Release

#### 3.2.1. DOX-Loading Efficiency

UV-Vis measurements at 550 nm were used to quantify free DOX in the supernatant after binding. For a typical ratio of 10 µg·mL^−1^ DOX to an AgNP solution with an SPR-based concentration of ~0.3 mM silver atoms, loading efficiencies ranged from 50 to 80% across the different nanoparticle types. Green-synthesized AgNPs showed higher DOX loading (up to 80%), presumably due to the presence of multiple functional groups from gallic acid and possible hydrogen bonding sites. In contrast, triangular prisms sometimes require additional stabilizers (e.g., citrate or PVP) that might compete with drug binding.

#### 3.2.2. pH-Sensitive Release Profiles

**Figure 3:**
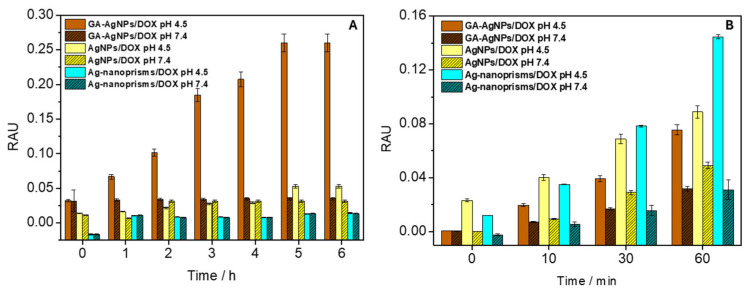
Evaluation of (**A**) DOX gradual release from AgNPs and (**B**) regulated ROS formation upon DOX loading on AgNPs surface tested in physiological and weakly acidic medium. RAU—relative arbitrary unit.

Figure 3A depicts the cumulative DOX release from AgNPs in PBS at pH 7.4 vs. pH 4.5, collected over 6 h at room temperature in dark conditions, using UV-Vis measurements at 550 nm. At pH 4.5, up to ~70% of DOX was liberated, whereas only ~15% was released at pH 7.4. Mechanistically, the acidic environment is believed to protonate amine groups on DOX, weakening electrostatic attractions to the nanoparticle surface. Simultaneously, the slight dissolution of AgNPs in mildly acidic media may facilitate additional drug desorption due to the protonation of DOX amino groups, which disrupts hydrogen bonds. The net effect is a triggered release that aligns well with tumor microenvironments, which often exhibit a pH 0.5–1.0 units lower than healthy tissues [42,43,44].

Drug release kinetics were also influenced by nanoparticle size and shape. Smaller spherical AgNPs tended to release cargo more rapidly, likely due to greater surface area-to-volume ratios. Triangular prisms, while capable of holding large drug payloads, exhibited a more gradual release. These results confirm the viability of tuning shape and size to match specific therapeutic regimens (fast release for acute dosing vs. sustained release for longer treatments).

#### 3.2.3. ROS-Mediated Cytotoxic Enhancement

Several references [17,45,46] highlight how partial dissolution of AgNPs or their direct catalytic activity can generate reactive oxygen species (ROS). This effect, in synergy with DOX—a known ROS inducer—could amplify tumor cell apoptosis. It remains essential, however, to manage the balance of ROS levels, as excessive oxidative stress can damage healthy cells. Preliminary cytotoxicity assays in our research suggested that DOX-loaded AgNPs had better tumoricidal selectivity than DOX alone, but in-depth mechanistic in vivo studies are still needed to confirm this synergy [33]. The impact of ROS formation in microenvironments with varying pH values was investigated to develop a pH-sensitive system for controlled drug release from the surface of individual AgNPs. The AgNPs/DOX nanocarrier mixture, along with NBD-Cl (as a ROS scavenger), was exposed to UV-A irradiation in a Petri dish for up to 60 min. After each exposure time, UV-Vis absorption spectra of NBD-Cl were recorded. As shown in Figure 3B, significantly higher ROS production (due to a more intense formation of NBD-Cl oxidation products at 470 nm) was observed for the AgNPs/DOX complex (notably higher for Ag-nanoprisms) at pH 4.5, which mimics the pH of endosomes in cancer cells, compared to the release at pH 7.4. The increased ROS formation is attributed to the combined oxidative activities of (a) DOX itself after detaching from the nanosurface, and (b) the AgNPs after releasing DOX, as both components have been shown to cause oxidative stress.

### 3.3. Biosensor Applications

**Figure 4:**
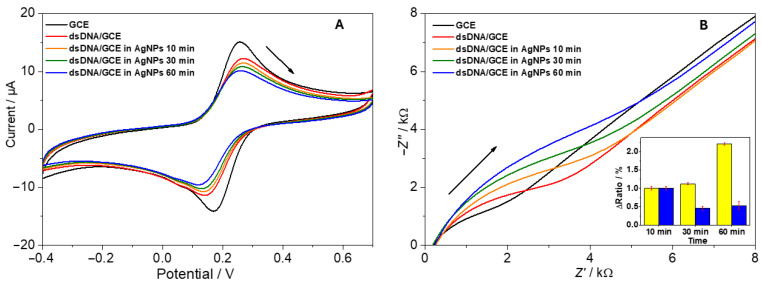
Electrochemical analysis of the AgNPs-dsDNA interaction using a dsDNA/GCE biosensor: (**A**) cyclic voltammograms and (**B**) Nyquist plots of 1 mM [Fe(CN)_6_]^3−^/[Fe(CN)_6_]^4−^. The inset represents the portion of survived salmon DNA determined using the biosensor and expressed Δ*R*_ct_ after the incubation of the DNA-based biosensors in NPs for a given time (yellow bars—AgNPs spherical, blue bars—AgNPs nanoprisms).

After building dsDNA layers on GCE surfaces, the subsequent incubation with AgNPs was expected to either hinder or facilitate electron transfer, depending on net charge, morphological changes, or partial conduction pathways. Figure 4A shows typical cyclic voltammograms recorded in 1 mM [Fe(CN)_6_]^3−^/[Fe(CN)_6_]^4−^:

- Clean GCE: This exhibited well-defined redox peaks with symmetrical anodic and cathodic profiles.
- dsDNA-modified GCE: This showed reduced peak currents due to electrostatic repulsion between the negatively charged phosphate backbone and negatively charged [Fe(CN)_6_].
- dsDNA + AgNPs (spherical): Additional blocking was observed after extended incubation caused by dsDNA and AgNPs electrostatic interaction; however, some electrons could tunnel through conductive AgNP pathways [33].
- dsDNA + AgNPs (nanoprisms): Notably, triangular nanoprism addition often displayed a slightly faster restoration of redox peak intensities, possibly owing to their larger, anisotropic surfaces that enhance electron bridging [47].

Electrochemical impedance spectroscopy (EIS) corroborated these findings, with Nyquist plots indicating an initial rise in charge transfer resistance (*R*_ct_) upon dsDNA deposition, followed by partial increases upon AgNP attachment, indicating a nanoparticle trapping onto the dsDNA structure. In the case of nanoprism-laden electrodes, the improved conductivity was more pronounced, evidenced by a reduction in *R*_ct_ values over time (Figure 4B, inset). *R*_ct_ values were obtained by a simulation of Randles circuit with the double-layer capacitance *C*_d_, the solution-phase resistance *R*_s_, and the Warburg impedance *Z*_W_. The normalized *R*_ct_ values were expressed as the ratio (Δ*ratio*) of the signal values before and after incubation of the biosensor with nanoparticles, relative to the value of the clean working electrode.

Similarly to our previous work [33], a sensitive DPV technique was selected to monitor two primary responses in the direct electrochemistry of nucleic acids: shifts in potential and changes in current upon the binding of guest molecules to the DNA structure. The dsDNA/GCE displayed DPV anodic peaks corresponding to deoxyguanosine (dGuo, at +0.90 V) and deoxyadenosine (dAdo, at +1.18 V). Following the incubation of the biosensor in the AgNPs solution, the peaks for dGuo and dAdo showed a gradual decrease over time. In relation to the CV and EIS results, where changes in charge transfer rate and resistance indicated the trapping of AgNPs by dsDNA, silver aggregates preferentially bind to the DNA phosphate backbone through electrostatic interactions. This results in a reduction in the current signal for dGuo and dAdo, suggesting the formation of an additional barrier by the trapped AgNPs that prevents nucleobase oxidation.

**Figure 5:**
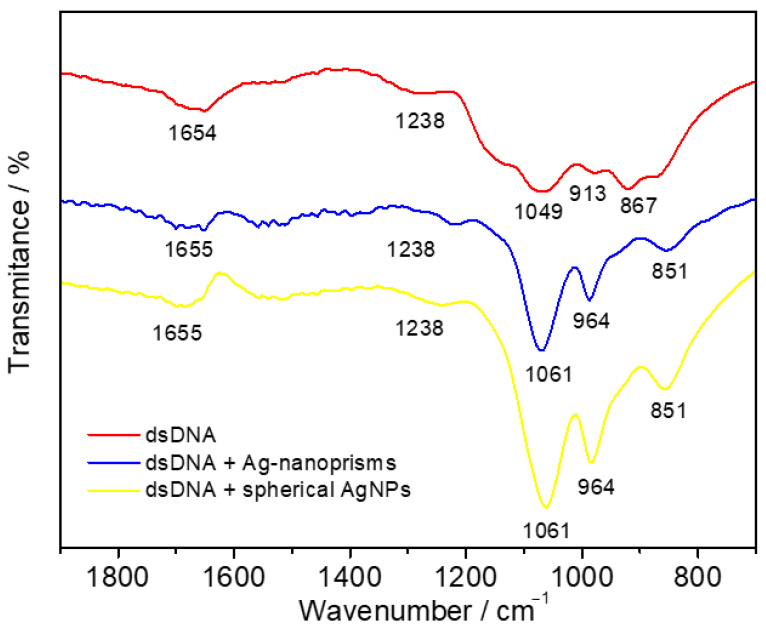
Spectral analysis of synthesized AgNPs and their interaction with dsDNA: FTIR spectra of dsDNA in the absence (red) and presence of Ag nanoprisms (blue) and spherical AgNPs (yellow) showing the electrostatic interaction with the DNA phosphate backbone.

FTIR analysis was performed to further explore the AgNPs-dsDNA interaction mechanism (Figure 5). IR analysis using Fourier transformation and an ATR crystal was conducted for single-drop analysis. Prior to analysis, the samples were left at room temperature for several minutes to allow interaction between dsDNA and individual AgNPs. Based on the literature [48], the absorption bands for dsDNA were identified as follows: 1694 and 1654 cm^−1^ for nucleobase C=O stretching, 1238 cm^−1^ for DNA phosphate asymmetric stretching, 1141 cm^−1^ for DNA phosphate symmetric stretching, 1049 cm^−1^ for C–O *D*-ribose stretching, 913 cm^−1^ for *D*-ribose ring vibration, and 867 cm^−1^ for C3′ endo-sugar stretching. After interaction with individual AgNPs, the nucleobase absorption bands remained unchanged, ranging from 1697 to 1655 cm^−1^. However, DNA phosphate bands in all cases shifted to higher wavenumbers (1049 to 1061 cm^−1^ and 913 to 964 cm^−1^), and the symmetric DNA phosphate stretching at 1141 cm^−1^ disappeared. These changes indicated a preferential electrostatic interaction between AgNPs and the DNA sugar-phosphate backbone.

## 4. Discussion

The synthesis and characterization of silver nanoparticles (AgNPs) in this study have provided valuable insights into their potential applications in drug delivery, biosensing, and reactive oxygen species (ROS)-mediated therapies. Through chemical reduction, green synthesis with gallic acid, and seed-mediated growth, we have generated AgNPs of varying sizes and shapes, each exhibiting distinct optical and electrochemical properties. These differences in morphology—spherical, green-synthesized, and triangular nanoprism—are likely to influence their interactions with biomolecules, their drug-loading capacity, and their overall therapeutic potential. However, while these results are promising, there are several important critical considerations that must be addressed in future studies, particularly in terms of scalability, biocompatibility, and environmental impact.

### 4.1. Synthesis and Characterization Challenges

While the green synthesis of AgNPs using gallic acid as both a reducing agent and capping agent offers an environmentally friendly alternative to chemical reduction, there are still challenges associated with the reproducibility of green synthesis methods. The presence of functional groups such as carboxyl and hydroxyl groups on the surface of the AgNPs can increase drug-loading efficiency, as seen with DOX, but also introduces variability in the synthesis process. Variations in the concentration of gallic acid, pH, and other reaction parameters may lead to inconsistent nanoparticle sizes or surface characteristics. These factors must be tightly controlled to ensure reproducibility for large-scale production [10].

Additionally, the seed-mediated growth method used to create triangular nanoprism AgNPs, while providing nanoparticles with larger surface areas for better drug retention, also introduces challenges regarding their stability. These nanoprisms are more prone to morphological changes under acidic conditions, which could limit their reliability in clinical applications [49]. Such morphological changes—such as shape transformation or dissolution—may also impact their biological interactions, possibly affecting their effectiveness and safety profiles. The potential instability of these larger nanoparticles in the acidic microenvironment of tumors could hinder their therapeutic potential, warranting further investigation into their long-term stability and behavior under physiological conditions.

### 4.2. pH-Sensitive Drug Release and ROS Generation

The pH-sensitive release profile observed in our study, particularly at pH 4.5, mimicking the acidic conditions of the tumor microenvironment, is promising for targeted drug delivery. However, it is essential to consider the balance between efficient drug release and the potential toxicity that could result from excessive ROS production. While ROS generation is beneficial for inducing apoptosis in cancer cells, excessive oxidative stress could harm surrounding healthy tissue. Therefore, precise control over ROS levels is critical. Further research should explore strategies to modulate ROS production, possibly through the use of antioxidants or by engineering nanoparticles with a “triggered” release mechanism that minimizes the risk of off-target toxicity [50].

Moreover, the dual action of DOX and AgNPs in generating ROS raises concerns about the cumulative oxidative stress they may produce in normal cells. While preliminary cytotoxicity assays suggest better tumoricidal selectivity with the AgNPs/DOX complex, in-depth in vivo studies are necessary to evaluate their real-world effectiveness and to determine whether the increased ROS production could cause unintended damage to healthy tissues [51]. The long-term implications of this toxicity need to be addressed before AgNPs can be considered for clinical use. The ability to fine-tune the ROS production while ensuring minimal impact on healthy cells should be a central focus in future investigations.

### 4.3. Environmental and Health Considerations

The environmental impact of synthesizing and utilizing AgNPs, particularly in large quantities, is an important issue that must not be overlooked. While green synthesis methods offer a more environmentally friendly alternative compared to conventional chemical reduction, the long-term effects of silver nanoparticles on ecosystems are still poorly understood. Silver is a heavy metal, and its release into the environment, particularly in wastewater from medical and industrial applications, could pose significant risks to aquatic life and human health. It is crucial to conduct environmental impact assessments to understand the potential for bioaccumulation and toxicity of AgNPs in natural environments [52,53].

Additionally, from a health perspective, the potential for AgNPs to accumulate in the human body after repeated exposure is a growing concern. Their interactions with cells, particularly within the liver, kidneys, and lungs, need to be thoroughly investigated to assess any long-term risks associated with nanoparticle accumulation. The development of AgNP-based therapies must consider the potential for chronic toxicity and the impact of nanoparticle degradation products, such as silver ions, on human health. Therefore, safety guidelines for the use of AgNPs in clinical settings must be established, and their biocompatibility, along with their clearance from the body, should be thoroughly studied [54,55].

### 4.4. Future Prospects

Despite these challenges, the potential applications of AgNPs in drug delivery and biosensing are vast. The ability to engineer nanoparticles with specific sizes and shapes, such as the green-synthesized spherical AgNPs or the seed-mediated triangular prisms, offers flexibility in developing targeted drug delivery systems. The dual action of AgNPs and DOX in generating ROS could be further optimized for combination therapies, where AgNPs act as both drug carriers and ROS generators, providing synergistic effects to combat cancer. The addition of biomolecules or targeting ligands to AgNPs could further enhance their selectivity and reduce off-target effects, improving their safety and efficacy in clinical applications.

In biosensor applications, the ability to fine-tune the morphology and surface characteristics of AgNPs will enable the development of highly sensitive and selective detection platforms for DNA, proteins, and other biomolecules. A promising future direction for this research is integrating functionalized DNA origami strands with AgNPs for electrochemical sensing. The utilization of DNA origami in biosensors has been demonstrated to offer highly specific recognition sites for target molecules, thereby enhancing the sensitivity and selectivity of the biosensors. In addition, the incorporation of AgNPs has been shown to facilitate pH-sensitive drug release, a process that is of significant interest in biomedical applications. The combination of these approaches has the potential to yield advanced multifunctional systems for targeted drug delivery and real-time disease monitoring. However, challenges related to stability, biocompatibility, and scalability must be addressed for the realization of clinical applications [56,57]. The electrochemical and optical properties of AgNPs can be exploited in a variety of sensing applications, from detecting biomarkers in early-stage diseases to monitoring drug release in real time.

## 5. Conclusions

AgNPs serve as efficient platforms for targeted drug delivery and biosensing. Their integration into smart systems promises advancements in precision medicine and diagnostics. By leveraging their unique optical and chemical properties, AgNPs enable real-time monitoring and tailored therapeutic delivery. The incorporation of green synthesis methods not only enhances biocompatibility but also supports sustainable development.

The results of this study underscore the versatility of AgNPs in addressing critical challenges in modern medicine. While challenges such as cytotoxicity and scalability persist, continued research efforts can refine these technologies for clinical use. The successful application of AgNPs in drug delivery and biosensors represents a step forward in the integration of nanotechnology into everyday healthcare solutions. Future innovations will likely focus on improving the multifunctionality and safety profile of AgNP-based systems, ensuring their widespread adoption in medical and diagnostic fields.

In conclusion, silver nanoparticles stand at the forefront of nanomedicine, offering unprecedented opportunities to improve diagnostics and treatment outcomes. Their adaptability, combined with advancements in synthesis and functionalization, positions them as a cornerstone of next-generation biomedical technologies.

## Figures and Tables

**Table 1:** The characteristics of AgNPs synthesized using different methods.

Synthesis Method	Shape	Size Range nm	SPR Peak nm
Chemical reduction	Spherical	10–20	420
Green synthesis	Spherical	15	410
Seed-mediated growth	Triangular prisms	50–100	750

## Data Availability

All data supporting the findings of this study are available within this article. Additional raw data and analytical method validation details are available from the corresponding authors upon reasonable request.
